# Non-autonomous Cellular Responses to Ototoxic Drug-Induced Stress and Death

**DOI:** 10.3389/fncel.2017.00252

**Published:** 2017-08-23

**Authors:** Shimon P. Francis, Lisa L. Cunningham

**Affiliations:** National Institute on Deafness and Other Communication Disorders, National Institutes of Health Bethesda, MD, United States

**Keywords:** ototoxicity, cisplatin, aminoglycoside, macrophages, glial cells, non-autonomous

## Abstract

The first major recognition of drug-induced hearing loss can be traced back more than seven decades to the development of streptomycin as an antimicrobial agent. Since then at least 130 therapeutic drugs have been recognized as having ototoxic side-effects. Two important classes of ototoxic drugs are the aminoglycoside antibiotics and the platinum-based antineoplastic agents. These drugs save the lives of millions of people worldwide, but they also cause irreparable hearing loss. In the inner ear, sensory hair cells (HCs) and spiral ganglion neurons (SGNs) are important cellular targets of these drugs, and most mechanistic studies have focused on the cell-autonomous responses of these cell types in response to ototoxic stress. Despite several decades of studies on ototoxicity, important unanswered questions remain, including the cellular and molecular mechanisms that determine whether HCs and SGNs will live or die when confronted with ototoxic challenge. Emerging evidence indicates that other cell types in the inner ear can act as mediators of survival or death of sensory cells and SGNs. For example, glia-like supporting cells (SCs) can promote survival of both HCs and SGNs. Alternatively, SCs can act to promote HC death and inhibit neural fiber expansion. Similarly, tissue resident macrophages activate either pro-survival or pro-death signaling that can influence HC survival after exposure to ototoxic agents. Together these data indicate that autonomous responses that occur within a stressed HC or SGN are not the only (and possibly not the primary) determinants of whether the stressed cell ultimately lives or dies. Instead non-cell-autonomous responses are emerging as significant determinants of HC and SGN survival vs. death in the face of ototoxic stress. The goal of this review is to summarize the current evidence on non-cell-autonomous responses to ototoxic stress and to discuss ways in which this knowledge may advance the development of therapies to reduce hearing loss caused by these drugs.

## Ototoxicity

Ototoxicity refers to damage to the inner ear by toxic substances. Some of these substances are medications that are used to treat a variety of conditions. It is estimated that 130–200 therapeutic drugs are ototoxic (Lien et al., 1983; Seligmann et al., 1996; Lanvers-Kaminsky et al., 2017). These include antibiotic glycopeptides and macrolides, anti-malarials, loop diuretics, and non-steroidal anti-inflammatory drugs (NSAIDs). Hearing loss caused by some of these drugs is reversible. However, there are two important classes of ototoxic drugs that cause permanent hearing loss: the aminoglycoside antibiotics and the platinum-based antineoplastic agents. The aminoglycoside antibiotics are effective for the treatment of a wide range of bacterial infections, including *Mycobacterium tuberculosis, Pseudomonas, Escherichia coli, and Klebsiella* (Schacht et al., 2012; Krause et al., 2016). Administration of clinically relevant doses of aminoglycosides results primarily in damage to basal turn OHCs in the cochlea and both type I and type II vestibular HCs (Tsuji et al., 2000; Hinojosa et al., 2001). Damage in the cochlea progresses in a gradient from base to apex, and in the vestibular maculae in a gradient from the striolar to the extrastriolar region. Prolonged or high-dose treatment with aminoglycosides also results in changes in the stria vascularis, including thinning, shrinkage, and atrophy (Forge and Fradis, 1985; Forge et al., 1987). While many cell types in the inner ear internalize aminoglycosides, the HCs are particularly vulnerable to aminoglycoside-induced death. Aminoglycoside-induced HC death has been described as both apoptotic and necrotic (Nakagawa et al., 1998; Lenoir et al., 1999; Matsui et al., 2002, 2003; Cunningham et al., 2004; Jiang et al., 2005). The molecular and cellular mechanisms that result in HC death are not fully understood, but there are a number of signaling molecules that are associated with aminoglycoside ototoxicity. One of the earliest observed indicators of toxicity is the formation of reactive oxygen species (ROS) in HCs (Priuska and Schacht, 1995; Hirose et al., 1997; Sha and Schacht, 1999). Increased oxidative burden in HCs and spiral ganglion neurons (SGNs) has been linked to activation of the c-jun-N-terminal kinase (JNK) stress signaling pathway, which in-turn has been shown to be associated with activation of pro-apoptotic caspases -8, -9, and -3, and HC death (Hirose et al., 1999; Pirvola et al., 2000; Cunningham et al., 2002, 2004; Matsui et al., 2002, 2003, 2004; Ylikoski et al., 2002; Cheng et al., 2003; Wang et al., 2003; Lee et al., 2004; Mangiardi et al., 2004; Sugahara et al., 2006; Jeong et al., 2010).

The platinum-based antineoplastic agents, cisplatin, hyphenate-oxali-platin, and carboplatin are among the most widely used anti-cancer drugs, and they are used to treat a variety of solid tumors in both pediatric and adult cancer patients. Cisplatin is the most commonly-used of these and is also the most ototoxic drug in clinical use (Muggia et al., 2015). As with aminoglycosides, cisplatin ototoxicity is associated with OHC loss in a basal to apical gradient, with the innermost row of OHCs being affected first (Schacht et al., 2012). Cisplatin-induced HC death is associated with oxidative stress, phosphorylation of STAT1, and activation of caspases -9 and -3 (Rybak et al., 2009; Schmitt et al., 2009). Reductions in both the endocochlear potential (EP) and compound action potential (CAP) are concomitant with OHC loss, indicating effects on the cells of the stria vascularis and SGNs (Schacht et al., 2012). *In vivo* studies indicate membrane blebbing and cytoplasmic vacuolization in strial marginal cells after cisplatin treatment, as well as shrinking and atrophy of intermediate cells, and demyelination, shrinkage, and apoptosis of SGNs (Cardinaal et al., 2000; Sluyter et al., 2003; van Ruijven et al., 2004; Ozkiris et al., 2013; Sun et al., 2016). Thus, unlike aminoglycosides, which primarily affect hair cells, cisplatin damages several cell types in the inner ear.

## Non-autonomous cellular disease pathologies

Like most diseases, drug-induced hearing loss has been studied in terms of the cells that are killed, primarily the HCs and SGNs, and the molecular and cellular signals specific to those cells. Rudolph Virchow is commonly referred to as the father of pathology, and his 1858 treatise on the “cell state” helped to form the basis of how we view diseases and their etiologies. Central to his dogma is the idea of cell autonomy. Virchow's vision of the cells of an organism was comparable with citizens in a society, with each cell having autonomy from the larger body (Reynolds, 2007). Since then, many diseases have been considered to have cell-autonomous etiologies and outcomes, with the assumption that pathology is caused by damage to a specific class of autonomous cells. Biologists now recognize that cells are a community, and the behavior of one cell type can influence whether another cell type lives or dies after stress (Cleveland and Rothstein, 2001; Barbeito et al., 2004; Kemp et al., 2011; Popiel et al., 2012; May et al., 2013; Anttonen et al., 2016; Halievski et al., 2016; Macrez et al., 2016; Tognatta and Miller, 2016; Olson et al., 2017). Studies of CNS diseases have provided insights into non-autonomous cell death. Stress or injury in CNS neurons can induce phenotypic changes in both astrocytes and microglia (known as reactive astrocytosis or gliosis, respectively) that can either exacerbate or limit the extent of progressive neuronal cell death (Cleveland and Rothstein, 2001; Barbeito et al., 2004; Compston and Coles, 2008; Ilieva et al., 2009; Macrez et al., 2016; Kipp et al., 2017). Thus, glial cells are often mediators of whether a neuron under stress will live or die.

Examples of non-cell-autonomous pathologies are associated with many glial cell types of the CNS. Amyotrophic lateral sclerosis (ALS) is a neurodegenerative disease that results in progressive muscle deterioration and paralysis. This pathology is attributed to the death of motor neurons and activation of glial cells in the lumbar spinal cord (Cleveland and Rothstein, 2001; Rowland and Shneider, 2001; Barbeito et al., 2004; Vargas and Johnson, 2010). Several studies suggest that while the onset of ALS may begin in neurons, disease progression is due to reactive astrocytosis (Cleveland and Rothstein, 2001; Rowland and Shneider, 2001; Barbeito et al., 2004). These reactive astrocytes downregulate trophic machinery, such as glutamate transporters, and they induce the expression of pro-inflammatory cytokines (Cleveland and Rothstein, 2001; Rowland and Shneider, 2001; Barbeito et al., 2004). These changes result in deterioration of otherwise healthy motor neurons, initiating a positive feedback cascade and disease progression. Similarly, autism spectrum disorder (ASD) refers to a group of developmental disorders associated with abnormalities in language and social interaction. The etiology of ASD is still unknown, but there is an increasing body of research that suggests a link to defective microglia, abnormal synaptogenesis, inflammatory gliosis, and destruction of CNS connectivity by reactive microglia (Vargas et al., 2005; Bessis et al., 2007; Morgan et al., 2010; Suzuki et al., 2013). Finally, multiple sclerosis (MS) is a common autoimmune disease that is characterized by chronic inflammation and demyelination, resulting in motor, sensory, and cognitive defects (Compston and Coles, 2008; Domingues et al., 2016; Macrez et al., 2016; Kipp et al., 2017). MS arises from a nexus of non-cell-autonomous interactions between the adaptive (T and B cells) and innate (microglia) immune systems, along with astrocytes and oligodendrocytes (Kotter et al., 2006; Compston and Coles, 2008; Domingues et al., 2016; Duncan and Radcliff, 2016; Macrez et al., 2016; Tognatta and Miller, 2016; Kipp et al., 2017). Autoimmune responses of T and B cells result in activation of microglia and/or astrocytes, resulting in inflammation and possibly glutamate toxicity that converge on oligodendrocytes and ultimately neurons (Domingues et al., 2016; Duncan and Radcliff, 2016; Macrez et al., 2016; Tognatta and Miller, 2016). Thus, CNS diseases can be driven by non-autonomous cellular mechanisms in which non-neuronal cell types promote degeneration of neurons. Similarly, hearing loss is not simply a manifestation of autonomous HC and SGN dysfunction. Recent studies indicate that it is important to also consider the effects of ototoxic drugs on other cell types of the inner ear, including SCs and macrophages, and that these cells are critical determinants of recovery (or death) of sensory cells and neurons after ototoxic injury (Bichler et al., 1983; Sugawara et al., 2005; Ladrech et al., 2007; Lahne and Gale, 2008; Sato et al., 2010; May et al., 2013; Baker et al., 2015; Sun et al., 2015; Takada et al., 2015; Anttonen et al., 2016; Jadali and Kwan, 2016; Kim et al., 2016).

## Supporting cell functions in the undamaged inner ear

Supporting cells of the inner ear are analogous to glial cells of the central nervous system, expressing glial markers, such as vimentin, glutamate-aspartate transporter (GLAST), and glial fibrillary acidic protein (GFAP) (Anniko et al., 1986; Furness and Lehre, 1997; Rio et al., 2002). Like glial cells, SCs also play important non-cell-autonomous roles by promoting either survival or death of HCs and/or SGNs in the inner ear during and after ototoxic challenge. Supporting cells are highly specialized and polarized accessory epithelial cells that support the functions and viability of sensory HCs and neurons by providing structural integrity, trophic factors, and potassium and glutamate recycling. The mammalian organ of Corti contains at least seven different SC types: Deiters' cells, pillar cells, Hensen's cells, inner phalangeal cells, inner border cells, Claudius' cells, and Boettcher cells in the basal turn. All of these supporting cell types are required for normal hearing function (Wan et al., 2013; Burns et al., 2015).

Supporting cells are integral to the maintenance of the integrity of the reticular lamina, ion recycling, and thus normal functioning of the inner ear. The inner ear consists of three fluid-filled compartments, each with different ionic compositions. Endolymph in the scala media is high in potassium and low in sodium, and it bathes the apical surfaces of the organ of Corti. Perilymph in the scala vestibuli and scala tympani is low in potassium and high in sodium, and it bathes the basolateral surfaces of HCs and SCs. The electrochemical gradient produced by the separation of these fluid compartments is essential for HC mechanotransduction and normal hearing (Wangemann, 2006). The reticular lamina provides an impermeable epithelial barrier between the endolymph and perilymph. This structure is formed by a network of special “very tight” junctions between HCs and their neighboring SCs (Anniko and Wroblewski, 1986). In the event of HC degeneration and death, SCs seal the resulting wound, maintaining the integrity of the reticular lamina (Forge, 1985; Cotanche and Dopyera, 1990; Li et al., 1995; Hordichok and Steyger, 2007; Anttonen et al., 2014). In addition to providing structural and trophic support to HCs, SCs also express neurotrophins and neurotrophin receptors that are required for the long-term survival of SGNs (Stankovic et al., 2004). Thus, supporting cells are specialized glia that promote the functions of both hair cells and SGNs and are required for hearing function.

## Supporting cells and hair cell death

In the event of HC death and degeneration, a critical response of SCs is rapid expansion to fill the voids left by degenerating HCs in order to maintain the integrity of the reticular lamina (Forge, 1985; Cotanche and Dopyera, 1990; Li et al., 1995; Hordichok and Steyger, 2007; Anttonen et al., 2014). Ototoxic damage to HCs has the potential to disrupt the reticular lamina, reducing the EP, and potentially exacerbating the injury by exposing the lateral membranes of HCs and SCs to high levels of potassium in endolymph (Cody et al., 1980; Bohne and Rabbitt, 1983; Meiteles and Raphael, 1994). Supporting cells preserve the integrity of the reticular lamina by either extruding or engulfing dead HCs and forming an epithelial scar (Forge, 1985; Cotanche and Dopyera, 1990; Meiteles and Raphael, 1994; Li et al., 1995; Hordichok and Steyger, 2007; Bird et al., 2010; Anttonen et al., 2014; Monzack et al., 2015). The mode of HC clearance (extrusion vs. phagocytic engulfment) varies among species, and even between the auditory and vestibular systems within the same species (Dodson et al., 1982; Li et al., 1995; Nakagawa et al., 1997). Extrusion is the predominant mode for removal of dead hair cells in the chick auditory system and guinea pig vestibular system (Cotanche and Dopyera, 1990; Li et al., 1995; Mangiardi et al., 2004). HC extrusion is usually preceded by ballooning of the entire apical surface of the HC; this is followed by ejection of the entire HC with the stereocilia bundle generally still attached (Hirose et al., 2004; Mangiardi et al., 2004). In addition to dead HCs, it appears that even viable HCs may be extruded, since some extruded HCs maintain membrane integrity (Mangiardi et al., 2004). Scanning electron microscopic analysis of the vestibular organs of guinea pigs injected with aminoglycosides reveals that HC bodies are extruded from the sensory epithelium, and SCs rapidly expand apical processes that forms a scar and seals the reticular lamina (Li et al., 1995).

The second mode by which supporting cells remove dead hair cells and preserve the reticular lamina is sub-luminal phagocytosis, which is the dominant means of apoptotic HC clearance in the chick and most mammalian vestibular organs and the mammalian cochlea (Forge, 1985; Bird et al., 2010; Anttonen et al., 2014; Monzack et al., 2015). In this process, dead HCs are engulfed by neighboring SCs. In the chick and mouse utricle this HC removal process occurs as two distinct events, an initial excision of the stereocilia bundle and cuticular plate and a second phagocytic engulfment of the remainder of the HC body (Bird et al., 2010; Monzack et al., 2015). SCs initiate excision of the stereocilia bundle by extending a cable-like circumferential actin belt at the apical portion of the dying HC. Constriction of this actin belt results in the excision of the cuticular plate and hair bundle, which either remain attached to the epithelium or are ejected into the lumen. This process also results in the formation of an epithelial scar (Li et al., 1995; Bird et al., 2010; Monzack et al., 2015). The second phase is characterized by SC extension of an actin-based phagosome that engulfs the remaining HC soma (Bird et al., 2010; Monzack et al., 2015). The HC appears to be degraded within this phagosome. In the mammalian cochlea, the phalangeal processes of Deiters' cells expand below the cuticular plate, excising the stereocilia bundle and engulfing the bodies of dead HCs (Forge, 1985; Anttonen et al., 2014). Whether via extrusion or phagocytosis, SCs play critical roles in the removal of dead HCs, thus preserving the integrity of the reticular lamina and sensory epithelium.

## Non-autonomous cellular responses to ototoxic drug-induced hair cell stress: responses of supporting cells

**Figure 1:**
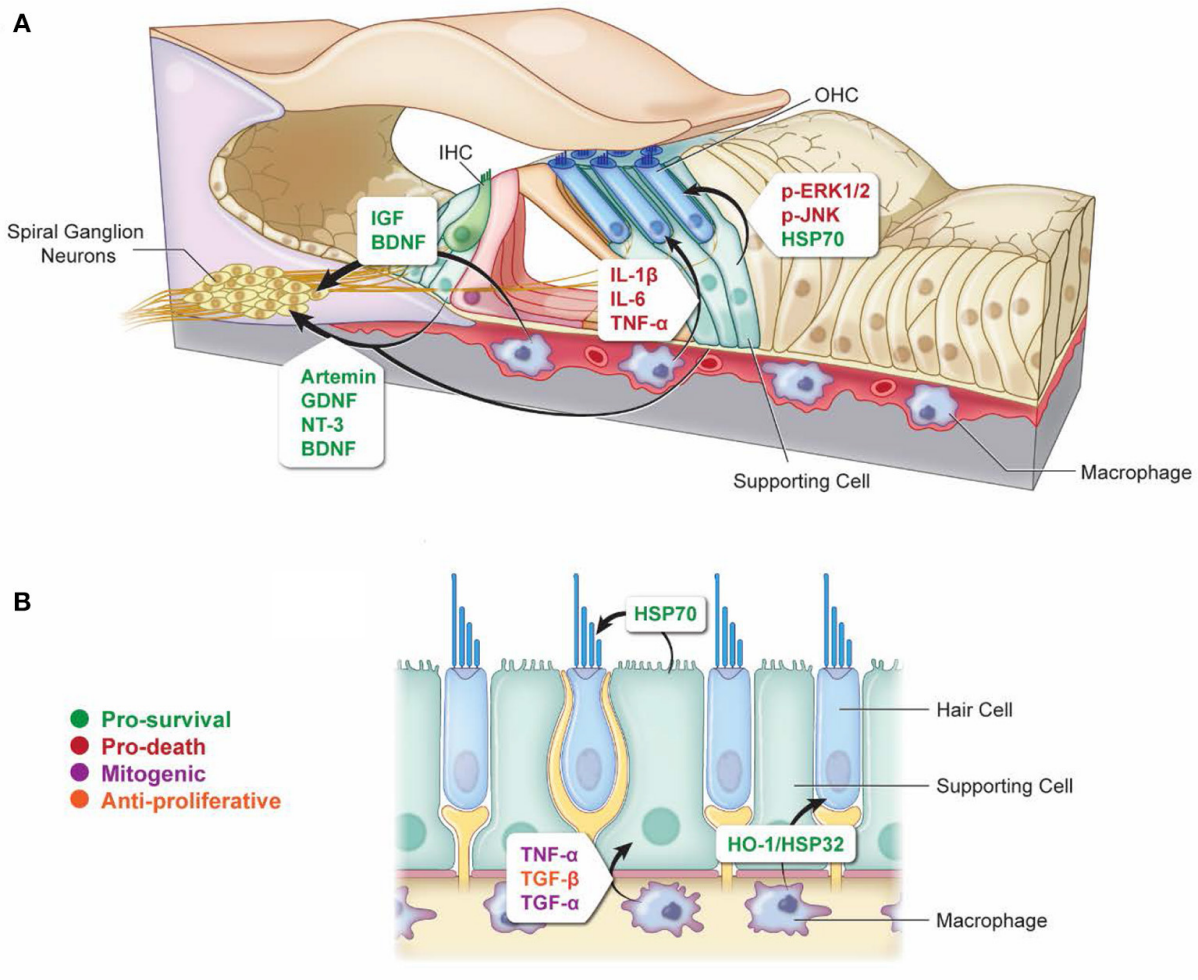
Non-autonomous cellular signals influence survival of HCs and SGNs as well as SC proliferation after ototoxic insult. **(A)** In response to ototoxic stress in the cochlea, SCs can promote either survival (via induction of HSP70) (Takada et al., 2015) or death (via activation of ERK1/2 and JNK pathways) (Lahne and Gale, 2008; Anttonen et al., 2016) of HCs. Supporting cells can also support the survival of SGNs after ototoxic injury through the release of protective neurotrophins (Sugawara et al., 2007; Zilberstein et al., 2012; Bailey and Green, 2014). Similarly, macrophages can promote survival of SGNs by releasing cytoprotective neurotrophins (Kaur et al., 2015b), and they can also promote HC death after ototoxic injury by releasing pro-inflammatory cytokines (Sato et al., 2010; Sun et al., 2015). **(B)** Vestibular SCs and macrophages can promote HC survival during ototoxic stress through the release of HSP70 and HO-1/HSP32, respectively (May et al., 2013; Baker et al., 2015). Macrophages can promote SC proliferation in response to ototoxic stress through the release of mitogenic cytokines TNF-α and TGF-α, or they can suppress SC proliferation through release of TGF-β (Warchol, 1999). Arrows indicate which cell types are involved in each signaling event. Signaling molecules are indicated beside each arrow. Green, pro-survival signals; Red, pro-death signals; Purple, mitogenic signals; Orange, anti-proliferative signals.

Supporting cells play central roles in mediating HC survival and death after damage (Figure 1). Exposure to aminoglycosides results in SC-specific activation of ERK1/2 mitogen-activated protein kinase (MAPK) in neonatal rat cochlear explants (Lahne and Gale, 2008). ERK1/2 activation begins in SCs at the site of injury and spreads to adjacent SCs in the immediate region in a manner that depends on inter-cellular communication via gap junctions. Pharmacological inhibition of ERK1/2 signaling reduces aminoglycoside-induced HC death (Lahne and Gale, 2008). These data indicate that ERK1/2 is activated specifically in SCs in response to aminoglycoside ototoxicity, and that this activation promotes hair cell death. Thus, the factors that determine whether the aminoglycoside-exposed HC will live or die are not entirely autonomous to the hair cell. ERK-dependent signals from SCs can promote non-cell-autonomous HC death and thus determine the fate of HCs exposed to aminoglycosides.

The idea that SCs mediate the life vs. death fate of damaged HCs is further supported by *in vivo* data from the noise and aminoglycoside-damaged adult cochlea. Aminoglycoside ototoxicity results in activation of the c-Jun N-terminal protein kinase (JNK/c-Jun) pathway in hair cells, which is associated with ototoxic stress and cell death pathways (Pirvola et al., 2000; Ylikoski et al., 2002; Matsui et al., 2004; Sugahara et al., 2006; Francis et al., 2011, 2013; Anttonen et al., 2016). Inhibition of JNK signaling inhibits aminoglycoside-induced HC death both *in vitro* and *in vivo* (Pirvola et al., 2000; Ylikoski et al., 2002; Sugahara et al., 2006; Francis et al., 2013). Anttonen et al. (2016) observed an unexpected pattern of JNK activation in the organ of Corti of adult mice administered kanamycin (an aminoglycoside) and furosemide (a loop diuretic). Outer HC degeneration and apoptosis were accompanied by robust upregulation and phosphorylation of c-Jun (target of JNK activation) in cells that are not vulnerable to aminoglycoside-induced death, including IHCs and SCs, especially Deiters' cells. In contrast, OHCs were negative for both c-Jun expression and phosphorylation (JNK activation). OHCs are required for the damage-associated JNK activation in SCs, and blockade of JNK activation specifically in SCs resulted in partial protection against acoustic trauma. These data suggest that JNK activation in SCs may reflect damage signaling from HCs under stress. In addition, JNK activation in SCs may mediate OHC degeneration (Anttonen et al., 2016). Together these results indicate that stress signaling in SCs can promote HC death *in vivo*.

Non-cell-autonomous signaling from SCs may also influence HC death in response to cisplatin ototoxicity. The gap junction protein connexin 43 is expressed in SCs and not in HCs, yet it can function as a pro-apoptotic mediator of cisplatin-induced HC death (Kikuchi et al., 1995; Zhao and Santos-Sacchi, 1999; Zhao and Yu, 2006; Zhao et al., 2006; Yu and Zhao, 2009; Kim et al., 2016). Inhibition of gap junction signaling reduced cisplatin-induced hearing loss, as measured by auditory brainstem response (ABR), suggesting that SC signaling via gap junctions may promote cisplatin-induced hair cell death (Kim et al., 2016). Gap junctional intercellular communication may either promote cell survival or cell death, depending on the context of injury. Connexin hemi-channels can be conduits for ATP release, and it is reasonable to consider the possibility that SC intercellular communication can either enhance or inhibit the spread of an ototoxic insult (Lahne and Gale, 2008).

In addition to promoting HC death, SCs can also protect HCs undergoing ototoxic challenge. Induction of heat shock proteins (HSPs) is protective against both aminoglycoside- and cisplatin-induced HC death in adult mouse utricle explants (Cunningham and Brandon, 2006; Taleb et al., 2008, 2009; Francis et al., 2011; May et al., 2013). Following heat shock, inducible HSP70 is localized primarily in SCs, with little immunoreactivity detected in HCs, suggesting that SCs mediate the protective effect of HSP70 induction. Supporting-cell-specific expression of HSP70 by adenovirus (AdHSP70) is also protective against aminoglycoside-induced HC death, indicating that HSP70 expression in SCs is sufficient to protect HCs from aminoglycoside ototoxicity (May et al., 2013). SCs may confer their protective effect by secreting HSP70 into the extracellular environment (May et al., 2013). In support of this hypothesis, extracellular HSP70 is required for the protective effect of heat shock (May et al., 2013). These data indicate that non-cell-autonomous signaling from SCs can promote survival of HCs treated with aminoglycosides *in vitro*. Evidence for the protective effect of SC-derived HSP70 *in vivo* comes from a study in which cochleas of adult guinea pig were infected with AdHSP70, resulting in robust HSP70 immunoreactivity in Deiters' cells and pillar cells, with no immunoreactivity observed in HCs (Takada et al., 2015). When the animals were treated with kanamycin and furosemide to kill HCs, over-expression of HSP70 in SCs reduced IHC death and improved ABR thresholds in ears receiving viral HSP70 infection relative to untreated ears. OHCs were not protected against aminoglycoside-induced death, even though the SCs adjacent to them were infected by Ad-HSP70. It is possible that the magnitude of the damage caused by the combination of kanamycin and furosemide results in OHC damage that overwhelms HSP70-mediated protection. Regardless, both *in vitro* and *in vivo* data indicate that HCs can be protected against ototoxic drug-induced death in a non-autonomous manner by HSP expression in supporting cells.

The above-described data indicate that induction of protective molecules in SCs can promote HC survival after ototoxic insult. Taken together with the data indicating that SCs can also promote HC death (Lahne and Gale, 2008; Anttonen et al., 2016), a model emerges in which non-cell-autonomous signals are critical determinants of whether a HC under stress will ultimately live or die.

## Supporting cells and ototoxic drug-induced spiral ganglion neuron death: non-autonomous cellular responses

In addition to HCs, ototoxic drugs also cause death of SGNs and degeneration of peripheral auditory fibers (PAFs). Animal models and analysis of human temporal bones suggest that SCs influence the survival of SGNs and maintenance or regrowth of degenerated PAFs after ototoxic insult. Aminoglycoside-induced loss of OHCs in cats had little effect on degeneration the of SGNs and PAFs; however, loss of IHCs and their “supporting structures” (SCs) was followed by rapid retrograde degeneration of SGNs (Leake and Hradek, 1988). Systemic aminoglycoside administration in chinchilla revealed that changes in SC morphology were accompanied by degeneration of outer spiral afferent dendrites (Ryan et al., 1980). Even in instances of aminoglycoside ototoxicity that result in near complete loss of both IHCs and OHCs, there was significantly more neuronal survival in areas where SCs remained intact (Sugawara et al., 2005). Studies of human temporal bones confirm that even in cases of severe HC loss, 5–10% of SGNs “resist retrograde degeneration” (Spoendlin, 1975). Schuknecht was one of the first to identify the correlation between surviving SCs and the presence of surviving PAFs (Schuknecht, 1953). Analysis of temporal bones from patients with a variety of inner ear pathologies including exposure to ototoxic drugs indicated that peripheral fibers were able to survive in the absence of IHCs and OHCs, but not in the absence of intact SCs (Johnsson, 1974; Otte et al., 1978; Johnsson et al., 1981; Suzuka and Schuknecht, 1988). The loss of these SCs was thought to lead to retrograde degeneration of PAF and SGNs, and the extent of SGN degeneration was directly correlated to the extent of SC loss (Suzuka and Schuknecht, 1988). Together these studies suggest that SCs (but not HCs) must be present in order for PAFs and SGNs to survive.

In contrast to the above studies suggesting that SGN survival requires SCs, there are also studies suggesting that intact differentiated SCs are not required for peripheral auditory fiber or SGN survival (Bichler et al., 1983; Leake and Hradek, 1988). These conflicting reports may be related to the identity of the cells that remain in the undifferentiated “flat” epithelium observed after severe ototoxicity. Are these cells de-differentiated SCs, or possibly cells from adjacent regions that have migrated to replace dead SCs (Kim and Raphael, 2007; Izumikawa et al., 2008; Oesterle and Campbell, 2009; Abbas and Rivolta, 2015)? Studies by Abbas and Rivolta (Abbas and Rivolta, 2015) suggest that the cells making up the flat epithelium are SCs. Although these cells have lost their characteristic morphologies, they continue to express SC markers, including OCP2 and acetylated αTubulin. Both IHCs and SCs provide SGNs with neurotrophic factors that are required for their survival (Ernfors et al., 1992; Farinas et al., 2001; Sobkowicz et al., 2002). NT-3 expression in SCs is required for the survival of SGNs in both the cochlea and vestibular organs (Sugawara et al., 2007). In the adult cochlea deafened by aminoglycosides, NT-3 is expressed primarily by surviving, differentiated SCs (Bailey and Green, 2014). Additional neurotrophins are also expressed in the organ of Corti after aminoglycoside-induced HC death, suggesting that even in the absence of HCs, neurotrophic factors produced by SCs are available to SGNs (Bailey and Green, 2014). Moreover, genetic studies have shown that SGNs can survive in the absence of IHCs through development and into maturity, and this survival is dependent on neurotrophins provided by SCs (Zilberstein et al., 2012). These data suggest that the cells constituting the flat epithelium retain a SC phenotype, and these de-differentiated SCs continue to provide neurotrophic support to remaining SGNs and PAFs. Thus, in the studies suggesting that SCs were not required for survival of SGNs and PAFs, there may have been de-differentiated SCs present that were providing trophic support after ototoxic insult. Overall the data are consistent with a model in which supporting cells release trophic factors that are required for proper innervation, SGN and PAF homeostasis under normal conditions and after ototoxic insult (Figure 1).

## Non-autonomous cellular signaling from macrophages in the undamaged and lesioned inner ear

The inner ear was long believed to be an immune-privileged organ, like the brain and retina, due to the presence of tight junctions within the stria vascularis that constitute the blood-labyrinth barrier (BLB) (Harris, 1983, 1984; Fujioka et al., 2014). Early studies of inflammation after ototoxic insult indicated that macrophages did not infiltrate the organ of Corti and were only identified in the endolymphatic sac, along with cellular debris (Wright and Meyerhoff, 1984). Systemic administration of a highly immunogenic protein, keyhole limpet hemocyanin (KLH), resulted in very little antibody production in perilymph (Harris, 1983). In contrast, when perilymph was directly injected with KLH, there was a significant increase in anti-KLH titers, indicating that there is a resident population of immune cells (later identified as macrophages) within the inner ear (Harris, 1983, 1984). Therefore, it was hypothesized that these resident macrophages are capable of mounting an immune response, but the BLB serves as a barrier that blocks: (a) immune cells, (b) antigens from the inner ear that may provoke a systemic immune response, and (c) antibodies from systemic circulation (Harris, 1983, 1984). However, recent studies suggest that the inner ear is not an immune-privileged compartment; instead it is permeable to hematopoietic stem cells, both after injury and under normal conditions (Lang et al., 2006; Okano et al., 2008; Sato et al., 2010).

Macrophages are myeloid cells of the innate immune system, first described by Elie Metchikoff in the late nineteenth century and recognized for being highly phagocytic (Davies et al., 2013; Varol et al., 2015). Resident macrophages are present in the majority of tissues in the body, where they are integral to the maintenance of homeostasis through phagocytosis and degradation of dead cells, cellular debris, and foreign materials (Davies et al., 2013; Varol et al., 2015). Resident macrophages are present in the undamaged mammalian inner ear, where they perform functions required for the maintenance of the extracellular environment and the BLB (Hirose et al., 2005; Shi, 2010; Zhang et al., 2012; O'Malley et al., 2016). Some of the first studies into the interplay between macrophages and HCs suggested that they may play a role in wound healing and regeneration of lost HCs. After tail amputation in salamander, macrophages are recruited to the tissues surrounding the neuromast most proximal to the site of injury, and macrophage recruitment precedes the onset of SC mitotic activity in these neuromasts (Jones and Corwin, 1993). When neuromast HCs were damaged by laser ablation, macrophage infiltration peaked shortly before the onset of SC proliferation, suggesting that these macrophages may function to influence HC regeneration through the secretion of growth factors and mitogenic cytokines (Corwin et al., 1991; Jones and Corwin, 1996; Pei et al., 2016). Subsequent studies in chick utricle exposed to neomycin confirmed that cytokines secreted from activated macrophages influence the rate of SC proliferation (Warchol, 1999). These experiments suggest that macrophage activity is required for effective recovery of the sensory epithelium after injury, and macrophages have non-cell-autonomous effects on SC proliferation and regeneration of HCs after injury.

The undamaged chick auditory and vestibular organs contain a resident population of two distinct leukocyte-derived cells, identified as macrophages and “microglia-like” cells (MLCs), based on morphology and expression of the common leukocyte marker CD45 or monocyte marker CD68 (Bhave et al., 1998; Warchol, 1999). Macrophages (large round shape) are present immediately beneath the HC nuclear layer, whereas microglia-like cells are most often located in the SC layer. Numerous cells expressing macrophage markers (Bu-1, Cla, and 74.2) are also observed in regions outside of the sensory epithelium, such as the underlying stromal tissues (O'Halloran and Oesterle, 2004). After ototoxic injury, both macrophages and microglia-like cells are recruited to the sensory epithelium and luminal surface, suggesting that dying HCs attract them to sites of injury. In contrast, microglia-like cell numbers are increased in both damaged and undamaged regions (Bhave et al., 1998; Warchol, 1999). These data in conjunction with those on the release of mitogenic cytokines from macrophages (Warchol, 1999) suggest that macrophages/microglia-like cells are recruited to sites of ototoxic injury, where they may function to enhance the proliferation and/or differentiation of SCs after HC death in the avian inner ear.

Macrophages are also recruited to sites of ototoxic injury in the mammalian inner ear (Sato et al., 2010; Hirose and Sato, 2011; Sun et al., 2015). Potential roles for damage-recruited macrophages include phagocytosis of dead HCs and debris, and secretion of cytokines (Hirose et al., 2005; Sato et al., 2010; Kaur et al., 2015a,b; Sun et al., 2015). CD45+ macrophages were recruited to the tunnel of Corti as soon as the first week after systemic aminoglycoside administration in rats (Ladrech et al., 2007). The timing of macrophage infiltration coincided with the peak of HC death and ended when most of the OHCs and IHCs had been eliminated, suggesting that infiltration signals were mediated by HC death. This study provided no direct evidence for macrophage phagocytosis of apoptotic HCs or apoptotic bodies, but it suggested that macrophages may function in phagocytosis and/or wound repair. Transmission electron microscopic studies indicate that “microglia-like cells” are present in the rat organ of Corti after amikacin administration, and these cells contain endocytic vesicles filled with degenerated cellular material (Wang and Li, 2000). These data suggest that macrophages may assist SCs in the engulfment of dead HC debris after ototoxic insult. Further evidence for phagocytosis of dead HCs and HC debris by macrophages comes from studies using utricles from transgenic mice expressing the diphtheria toxin receptor (DTR) specifically in HCs (Kaur et al., 2015a). While these Pou4f3-huDTR mice are not a model of drug-induced ototoxicity, they do demonstrate some cellular changes that are similar to those that occur after ototoxic insult. CX3CR1-GFP (a leukocyte and/or macrophage-specific cell surface receptor that functions in macrophage recruitment) positive macrophages infiltrate the sensory epithelium in response to DT-mediated HC death. These macrophages appear to be recruited from the population of resident macrophages residing in the stromal tissues of the utricle (Kaur et al., 2015a). This increase in macrophage number is transient, suggesting that HC death was the driving force behind macrophage infiltration into the sensory epithelium. Macrophage processes were in frequent contact with potentially apoptotic HCs, and these macrophages were actively engulfing HC debris (Kaur et al., 2015a). Taken together these data indicate that a population of macrophages resides in the major compartments of the undamaged mammalian inner ear, and they are recruited to sites of ototoxic injury, where they may assist SCs in the phagocytosis of dead HCs and HC debris.

Macrophages may have functions beyond phagocytosis of HC corpses and debris after injury in the inner ear. Macrophage infiltration and signaling may mediate survival of HCs and SGNs after ototoxic insult. CX3CR1 KO mice show significantly more macrophage infiltration into the cochlea after aminoglycoside administration, compared to control and heterozygous mice (Sato et al., 2010). In addition, CX3CR1 KO mice are more susceptible to aminoglycoside-induced hearing loss and HC death. These studies indicate that CX3CR1 inhibits macrophage infiltration or cytokine secretion after ototoxic insult, and they suggest that macrophage infiltration can influence the extent of HC damage and hearing loss after ototoxic injury.

Further evidence for non-autonomous effects of macrophages on HC viability after ototoxic insult comes from examination of CX3CL1/CX3CR1 signaling during neomycin-induced HC death (Sun et al., 2015). CX3CL1 (fractalkine) is a chemokine located on the surface of neurons and endothelial cells, and it serves as a damage signal and ligand for CX3CR1 receptors on macrophages (Ransohoff et al., 2007). Systemic aminoglycoside administration in neonatal mice resulted in a significant increase in expression of the soluble form of CX3CL1 in HCs and induced migration and activation of macrophages or MLCs into the sensory epithelium. Exogenous application of CX3CL1 resulted in a significant increase in the number of MLCs in the sensory epithelium as well as increased pro-inflammatory cytokine secretion and increased HC death. Inhibition of MLC activation inhibited neomycin-induced IHC and OHC death and hearing loss, suggesting that cytokine production by activated MLCs exacerbates aminoglycoside-induced HC death (Sun et al., 2015). These data suggest that HCs communicate damage signals to macrophages through upregulation of soluble CX3CL1, and CX3CL1/CX3CR1 signaling is integral to macrophage recruitment after injury. In addition, macrophage recruitment and activation may exacerbate ototoxic lesions through inflammatory cytokine production (Figure 1).

Like SCs, macrophages also have the potential to promote survival of HCs after ototoxic injury. Treatment with an inducer of heme oxygenase 1 (HO-1, also called heat shock protein 32, HSP32) protected against cisplatin-induced hair cell death in utricle explants (Baker et al., 2015). HO-1 was induced only in resident macrophages with no induction in HCs or SCs. When macrophages were depleted from the explants, the protective effect of HSP32/HO-1 was abolished (Baker et al., 2015). These data indicate that HO-1 induction specifically in macrophages can protect hair cells against aminoglycoside-induced death. Thus, the protective effect of HO-1 induction against HC death is non-autonomous and is mediated by resident macrophages.

In addition to modulating the survival and death of HCs under stress, macrophages also modulate the survival of SGNs. Although it is not an ototoxic drug, diphtheria toxin injection in Pou4f3-huDTR/CX3CR1-GFP mice results in complete loss of OHCs and IHCs after 7 days, with no obvious effects on other cell types (Kaur et al., 2015b). Recruitment of resident macrophages was significantly increased in the cochlea after DT injection. Infiltrating macrophages were observed below the basilar membrane and in Rosenthal's canal in association with SGN cell bodies. CX3CR1 KO mice had fewer macrophages infiltrating into the sensory epithelium and after DT injection compared with WT DTR mice. Notably, the reduction in macrophages within Rosenthal's canal was accompanied by a significant decrease in SGN survival after DT injection in CX3CR1 null mice. SGN numbers were unaffected in CX3CR1 KO mice that did not receive DT, suggesting that macrophages are only required for SGN survival after HC death. This is in contrast to previous studies showing an increase in macrophage infiltration in the cochleas of CX3CR1 KO mice administered aminoglycosides, which indicates that CX3CR1 and fractalkine signaling is complex, and maybe dependent on the cytotoxic insult or possibly the inner ear organ being analyzed (Sato et al., 2010; Kaur et al., 2015a). These data suggest that like SCs, macrophages can have either pro-death or pro-survival effects on both HCs and SGNs after injury, and these effects are likely mediated through the induction of cytotoxic cytokines, or protective molecules, such as HSPs and neurotrophins (Figure 1).

## Conclusion

Important non-cell-autonomous signals from surrounding cell types (SCs and macrophages) can increase or reduce the extent of ototoxic injury to both HCs and SGNs. Significant questions remain about these intercellular communication events. First, how do SCs and macrophages sense that HCs and SGNs are damaged? The damage signals that activate SCs and macrophages after ototoxic insult remain unclear; however, in other models of tissue damage and wound healing, transcription-independent signals are rapidly released from damaged cells, including calcium, ROS, and ATP (Lahne and Gale, 2008; Cordeiro and Jacinto, 2013). Second, how do SCs and macrophages differentiate between a “help me” signal and a “kill me” signal and thus decide whether to promote survival vs. death of damaged cells? Are these signals mediated by different molecules, or does the magnitude of the damage signal perhaps determine whether the SCs and macrophages act to promote death vs. survival? Finally, once a HC or SGN dies, what are the signals that mediate recognition of the dying cell for engulfment or extrusion (i.e., the “eat me” signal)? The importance of non-cell-autonomous signals in determining these life-or-death outcomes in the inner ear has only recently emerged, and it will be important to take these intercellular signaling events into account when developing therapies aimed at protecting hearing from the deleterious effects of ototoxic drugs.

## Author contributions

SF and LC conceived of and together outlined this review. SF wrote the manuscript. LC critiqued the manuscript.

### Conflict of interest statement

The authors declare that the research was conducted in the absence of any commercial or financial relationships that could be construed as a potential conflict of interest.
